# Scheme for Assisting Voluntary Hospitals

**Published:** 1920-05-22

**Authors:** 


### SCHEME FOR ASSISTING VOLUNTARY HOSPITALS.

One of the first public works of the Joint Council will be to help the voluntary hospitals in their present financial difficulties. The outline of the scheme, briefly described, is as follows:

1. To obtain a hospital survey showing the volume of work done during the year 1919 and the financial position in each individual hospital.
2. To make a public appeal for funds, £1,000,000 per annum is aimed at, and to allocate grants through a Grants Committee, on the recommendation of the Director of Hospital Services, after inquiry as to efficiency and needs.
3. To help in co-operative buying of hospital commodities.
4. To co-ordinate the hospitals into groups or counties or other areas.
5. To circulate statistical and other hospital information from the Central Bureau.
6. To relieve and help the hospitals by establishing more convalescent hospitals.

**Voluntary Hospital Assistance**—(*continued*).

7. To arrange meetings for the dissemination of knowledge, and to stimulate interest in all matters pertaining to public health.

Survey of 550 Hospitals in England and Wales.

As is generally known, the Director of Hospital Services under the Joint Council is Sir Napier Burnett, K.B.E., M.D. He has issued a general survey of the hospital situation throughout England and Wales, from which the above particulars have been taken. This survey does not include the hospitals of the Metropolis.

Sir Napier Burnett, in referring to the meeting held at St. Thomas's Hospital on January 23, 1920, is incorrect in describing it as "a meeting of the secretaries and house governors of the provincial hospitals." It was a meeting convened by the British Hospitals Association, and of the many present a large proportion were treasurers, members of committee, and secretaries of hospitals in London. At this meeting it was decided to accept the assistance of the Joint Council, and to ask them, in the first place, to give their attention to the provincial hospitals, which were, it was thought, more badly in need of assistance than the hospitals of London.

The survey deals with 550 hospitals in the fifty-five counties of England and Wales, which are stated to be approximately 78 per cent. of the voluntary civil hospitals (Burdett's "Hospitals and Charities" gives particulars of 581 hospitals in the same area), and omits those dealing exclusively with tuberculosis. The data of the work done in 1919 does not appear to be complete, but 507 of the hospitals are stated to have 29,821 available beds, and 498 treated 350,459 in-patients during the year, while 376 treated 1,600,869 out-patients. 33,500 cases (? of in-patients) are classified as medical and surgical, the former numbering 7,034, or 21 per cent. of the total, and the latter 26,466, or 79 per cent. of the total.

Gross Financial Position of 543 Hospitals.

543 hospitals showed, during 1919, ordinary income £2,835,269, and ordinary expenditure £3,310,896, the excess of expenditure over income being £475,627.

Of the ordinary income of 316 hospitals, £451,426 is shown as workmen's contributions. In 464 hospitals, £214,570 is shown as patients' contributions. In 248 hospitals £517,890 is shown as Public Services (War Office, Pensions Ministry, Borough or County Councils), and in 512 hospitals £423,044 is shown as interest from investments. The total from these four sources, £1,606,930, equals 56.67 per cent. of the ordinary income of 543 hospitals. 449 hospitals show invested capital as £9,585,865.

In speaking of the question of selling securities for current needs attention is drawn to the great depreciation in value, and of the fact that hospitals cannot afford to unload their stock in the present state of the market.

Entering more into detail, the survey shows that by far the greater portion of the gross deficiency belongs to the larger hospitals; 82 of these (general hospitals of 100 beds and over) had together a deficit in 1919 of £355,685.

From the above it will be gathered that Sir Napier Burnett, who took the matter in hand less than four months ago, has done wonders in collecting sufficient information to throw a light upon the salient points of the situation. The survey is, of course, incomplete. This is not the fault of Sir Napier and his staff, but of those who, less prompt, have made incomplete or belated returns.

The Director's Comments on the Figures.

The survey, it is suggested, may be regarded as dealing with "the facts" of the situation. Two additional figures, not shown in the statement, were taken out, namely (*a*) the average length of stay of each in-patient, and (*b*) the average annual cost per occupied bed. The methods employed by some of the hospitals varied so much that there was no option but to discard the details in both cases.

The Director alludes to the preponderance of surgical cases, and thinks that the gradual exclusion of medical patients is a matter of some importance, although possibly these cases may be better treated in the quieter atmosphere of the convalescent home rather than amidst the turmoil of a large town or city.

The chief difficulties experienced at present are lack of financial support, of bed accommodation, and of nurses. To relieve these difficulties the scheme outlined above has been prepared. The Director hopes that some such steps will assist in the development of a National Co-ordinated Hospital System for the cure and prevention of disease.

"Just as the two Societies of St. John and the Red Cross by their joint efforts did so much for the military soldiers both at home and in the various theatres of war during the past six years, so it is the intention and desire of the Joint Council to render similar service to the industrial soldiers at home, both men and women." With these words Sir Napier Burnett concludes his survey, upon the manner and matter of which he is to be heartily congratulated. We hope to publish his survey in full in a succeeding issue.

